# Presence of alternative lengthening of telomeres associated circular extrachromosome telomere repeats in primary leukemia cells of chronic myeloid leukemia

**DOI:** 10.1186/1756-8722-6-26

**Published:** 2013-04-02

**Authors:** Oumar Samassekou, Abba Malina, Josée Hébert, Ju Yan

**Affiliations:** 1 Division of Genetics, Department of Pediatrics, Faculty of Medicine and Health Sciences, Université de Sherbrooke, 3001, 12th Avenue North, Sherbrooke, QC J1H 5N4, Canada; 2 Department of Biochemistry, McGill University, 3655 Promenade Sir William Osler, Montreal, QC, H3G 1Y6, Canada; 3 Leukemia Cell Bank of Quebec, and Division of Hematology-Oncology, Maisonneuve-Rosemont Hospital, 5415 Boul. de l’Assomption, Montréal, QC, H1T 2M4, Canada

**Keywords:** CML, Telomeres, Cancer biology

## Abstract

**Background:**

The predominant mechanism by which human tumors maintain telomere length is via telomerase. In ~10% of tumor samples, however, telomere length is conserved, despite no detectable telomerase activity, in part through activation of the alternative lengthening of telomeres (ALT) pathway.

**Methods:**

We studied the circular extra-chromosomal telomeric repeat (ECTR), an ALT hallmark, and telomerase activity in 24 chronic myeloid leukemia (CML) patients in chronic phase (CP).

**Results:**

We identified the presence of ECTR in primary leukemia cells from some of these samples, which indicates the possible involvement of an ALT mechanism. Moreover, we found that some samples exhibited both circular ECTR and telomerase activities, suggesting that both mechanisms can contribute to the onset of CML.

**Conclusion:**

We propose that ALT or the combined activities of ALT and telomerase might be required for the early stages of leukemogenesis. These findings shed new light into the oncogenic pathways responsible for the maintenance of telomere length in leukemia, which will ultimately determine the effectiveness of anti-telomerase-based treatment protocols.

## Introduction

Human telomeres are long tandem arrays of TTAGGG bases associated with specialized nucleoprotein complexes localized at the physical ends of linear eukaryotic chromosomes that maintain the stability and the integrity of chromosomes [1]. Normal somatic cells undergo a finite number of cell divisions. Each successive round of replication progressively shortens telomeres and once they reach a critical length they trigger a permanently differentiated cellular state termed replicative senescence. When cells become “transformed”, either by mutations or by viral oncogenes, the mechanisms limiting cell replication become inactivated and, as a consequence, telomeres shorten beyond a stable threshold, pushing these cells into crisis and death. Those cells that do survive and continue to replicate now have activated telomerase, an RNA-dependent DNA polymerase, or an alternative lengthening of telomeres (ALT) mechanism to specifically maintain telomere length and stabilize chromosome ends. ALT occurs in about 10% of human tumors [2] and uses homologous recombination (HR) mediated replication to specifically elongate telomeres, which often results in different sizes of telomeres [3]. The use of HR by ALT positive cells can result in several cellular abnormalities which are considered “hallmarks” of activation of the pathway, including: 1) ALT-associated promyelocytic leukemia bodies (APBs), which are present in 5-35% of ALT cells [4], 2) telomere length heterogeneity ranging from 2 kb to greater than 20 kb in length [4,5], and 3) the appearance of circular ECTR [6,7], the latter of which is considered to be most reliable of all of the markers [2]. While recombination-mediated ALT is thought of as the predominant mechanism that cells generate circular ECTRs, they can also arise from exposure to DNA damaging agents [8], overexpression of the telomerase catalytic subunit via telomere length trimming mechanism [9], or through catastrophic double-strand breaks that arise from overexpression of a truncated mutant of TRF2 lacking its basic domain [10].

Chronic myeloid leukemia (CML) is a malignant and clonal hematopoietic stem cell disorder characterized by overproduction of myeloid cells and a specific cytogenetic marker, the Philadelphia (Ph) chromosome, which results from a reciprocal translocation between chromosomes 9 and 22, t(9;22)(q34;q11.2) [11]. Without effective therapy, CML irremediably progresses through three phases: chronic (CP), accelerated (AP), and blast crisis (BP) phases. Like most cancer cells, the average length of telomeres of CML cells are shorter than that of normal cells and this telomere shortening is more pronounced during disease progression [12], despite the fact that in the later stages of CML telomerase is highly expressed [13]. In our recent study on individual telomere length in CML, we found that although global telomere lengths in CML patient samples were shorter than normal, they do harbor distinctly long chromosome-specific telomeres [14], suggesting a unique signature of telomere length heterogeneity.

We hypothesized that this heterogeneity is probably the result of an active ALT pathway and not solely due to enhanced telomerase activity. In the present study, we show direct evidence for the presence of circular ECTR in CML, specifically the C-circles, indicating that ALT-mediated telomere regulation occurs in CML at a much higher frequency than previously thought.

## Results and discussion

**Figure 1:**
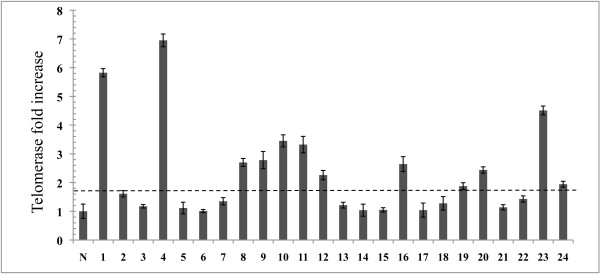
**Distribution of the level of telomerase activity in 24 CML samples. **Telomerase activity was measured by quantitative PCR (qPCR) in triplicate in CML samples. The average of telomerase activity from three normal samples (N) is used as a control to normalize against background level. Telomerase activity in CML is expressed as the fold-increase (*Y*-axis) to normal samples. Error bars indicate standard errors from average telomerase activity of three measurements for each tumor sample. Half of the samples fall into the high and another half of samples fall into the low telomerase activity according to the cutoff (dashed horizontal line).

**Figure 2:**
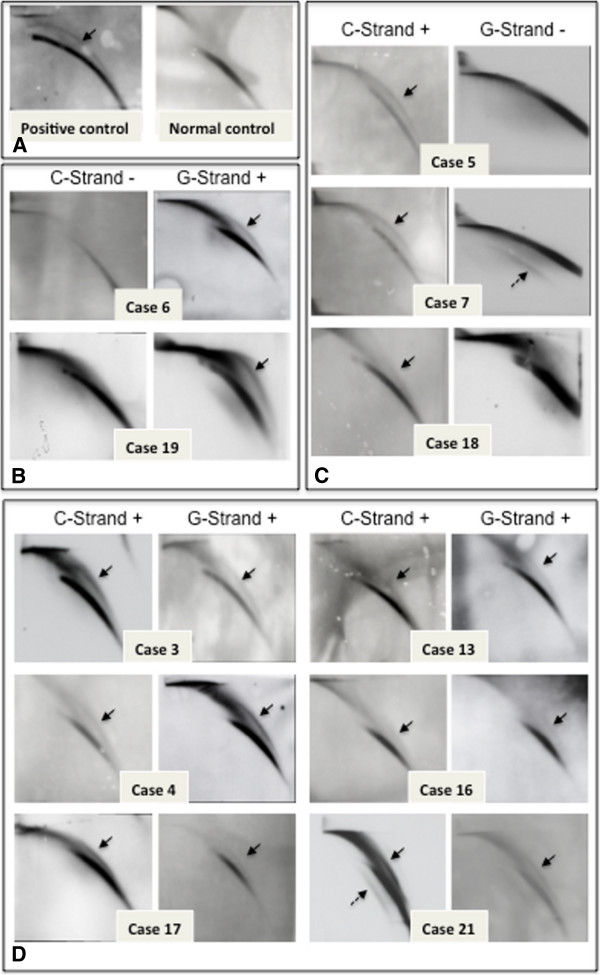
**Blots from 2D gels of telomeres showing the presence of ECTR molecules in CML samples.** (**A**) The presence and absence of the circular ECTR detected in C strand in a positive (left) and a negative (right) control sample. (**B**) Samples with circular ECTR in G-strand. (**C**) Samples with circular ECTR in C-strand. (**D**) Samples with circular ECTR in both C- and G-strands. Note that case 7 and case 21 also have linear ECTR molecules (dashed arrows).

Our recent study on the lengths of individual telomeres in CML patients in CP showed that despite significantly short average telomere length, some chromosome-specific telomeres in CML cells were longer and with distinct erosion rates than those in normal samples [14]. These findings suggested that the ALT pathway might be implicated in the maintenance of telomere lengths in these CML cells. We therefore assayed those same 24 CML samples for both telomerase activity and for circular ECTR, which is considered as one of the defining characteristics of the ALT activity. Note that all samples tested positive for the t(9;22)(q34;q11.2) translocation and that it was the only chromosomal abnormality in a majority (>95%) of the cells, indicating that virtually all of the cells were leukemic in origin. The level of telomerase activity in these 24 CML cases revealed that 12 (50%) had high telomerase activities (greater than 1.7 fold-difference), whereas the remaining samples showed no significant difference above normal background level, or had low telomerase activity (Figure 1 and Table 1). This proportion of samples presenting high telomerase activity is in agreement with other reports on telomerase activity in CML cells [15]. We then looked for circular ECTR by using 2D gel electrophoresis in these leukemia samples. A cell line with known activated ALT, GM0637 (Coriell Institute for Medical Research), was used as a positive control and peripheral white blood cells from three normal individuals were used as negative controls (Figure 2A). We found circular ECTR in 11 out of the 24 samples (46%) studied. Furthermore, we found that circular ECTR appeared in three forms in these 11 samples: single-G-stranded (2 samples) (Figure 2B), single-C-stranded (3 samples) (Figure 2C), and double-GC-stranded (6 samples) (Figure 2D). Two of these samples also displayed a single linear G-strand in addition to circular ECTR. Eight out of these 11 samples (73%) had concomitant low telomerase activity, while three samples (27%) displayed high telomerase activity (Figure 1).

**Table 1:** Summary of telomerase and 2D gel results in CML samples

		**Telomerase**		**Total**
		**+**	**-**	
Circles	CG	2	4	6
	G	1	1	2
	C	-	3	3
	**-**	9	4	13
Total		12	12	24

*CG*: Telomere double strand; *C*: Telomere C-single strand; *G*: telomere G-single strand.

ALT-dependent cells usually show little to no telomerase activity and have abundant ECTR in both linear and circular forms, especially C-stranded circles [2], caused by a t-loop deletion [10,15]. The presence of telomere C-circles in 3 out the 11 ECTR positive samples with no/low telomerase activity strongly indicates the use of ALT mechanism to maintain telomeres in these samples. Moreover, the five samples which displayed telomeric double-strand and G-strand circles with no/low telomerase activity might also suggest the ALT mechanism in these samples. Alternatively, the presence of telomere double-strand circles can also be due to dysfunctional TRF2, which can induce homologous recombination and lead to the resolution of the t-loop [10]. Furthermore, the presence of double-strand telomere circles with high telomerase activity might either be due to telomere uncapping defect in telomerase positive cells or the co-occurrence of both telomerase and ALT mechanisms in maintaining telomeres. The presence of both telomerase and ALT in telomere elongation appears to be a very rare phenomenon, but has been reported in only a few tumor types in general less than <5% of most tumor samples [16,17]. Nevertheless, in mouse models, the presence of an active telomerase did not prevent telomere recombination from occurring [18]. Therefore, it is feasible that these two mechanisms can act jointly or alternately to maintain telomere length in the same leukemia sample.

**Figure 3:**
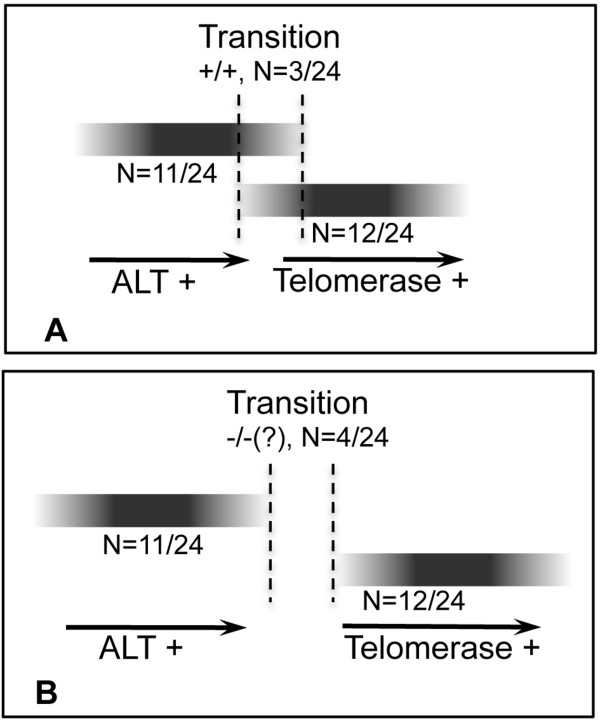
**Schematic depicting the transition between telomere maintenance mechanisms in CML samples.** (**A**) 3 out of 24 CML samples showed overlapping expression for both ECTR circles and telomerase activities at the transition state (between dashed vertical lines). (**B**) 4 out of 24 CML samples had neither detectable ECTR nor telomerase activity during the transition state (between dashed vertical lines). N: number of cases observed; +/+: double positive for telomerase and possible ALT mechanisms; -/-: double negative for telomerase and possible ALT mechanisms.

While telomeric C-circles are currently the most reliable of all the ALT markers [2], we cannot exclude the possibility that the samples where we could not detect ECTR are in fact positive in ALT, as we did not assay for other hallmarks such as ALT-associated promyelocytic leukaemia bodies (APBs) or telomere length heterogeneity. In fact, we might be underestimating the frequency of C-circles, as our assay of detection of ECTR, 2D gel electrophoresis, is probably less sensitive than recent methods based on rolling circle coupled with quantitative PCR [19]. Nevertheless, telomere recombination without detectable ECTR has been reported in some ALT-dependent cell lines [17] as well as in telomerase-deficient mice [16], making it feasible that ALT positive cells use a recombination-based mechanism without detectable ECTR. Indeed, such a case has been recently reported for mouse somatic cells specifically designed to detect for the synthesis of new telomeric DNA derived from a tagged telomeric template, allowing for the detection of ALT even in the absence of known ALT hallmarks [20]. With respect to those samples that were negative for both telomerase and ECTR, they could still be using the ALT mechanism to maintain their telomeres (our telomerase detection assay should be sensitive enough and those samples are not false-negatives). Alternatively, it is possible that those samples were simply assayed during an elongated transition phase between ALT and telomerase activation (Figure 3B).

Even though telomere lengths of typical ALT cell lines are heterogeneous, generating some very long telomeres (more than 20 kb) in some cases [10], most of our CML samples (including those with circular ECTR) were associated with short telomeres (an average of 6.64 kb) [14]. Although somewhat unusual, this phenomenon of homogeneously short telomeres in an ALT cell line has been previously documented [21]. Similarly, cells derived from the telomerase deficient mTR-/- mouse model have been shown to maintain their telomere lengths by a recombination-based mechanism without extensive telomere elongation as seen in many ALT cell lines [18]. Perhaps, these cells might use the same break-induced recombination mechanism to maintain their telomere lengths as seen in yeast type I telomerase-deficient survivor clones [22]. Furthermore, the recombination mechanism in this mouse model led to at least a five-fold difference in the lengths of telomeres on p- and q- arms of a given chromosome [18] which is what we have seen previously in these CML samples [14,23]. Thus, it seems likely that CML cells during CP might rely on a similar recombination mechanism as described in these different models in order to maintain their telomeres.

We proposed the following step-wise model to explain the transition from ALT to telomerase in the maintenance of telomeres during CML disease progression. In the early stages of CML leukemogenesis, ALT may act on a few specific chromosomes at first, perhaps by elongating telomeres on some key pro-growth or pro-survival chromosomes, conferring a cell proliferative advantage and thus leading to the formation of a leukemia cell clone [23]. With the progression of the disease, eventual activation of telomerase would take over telomere length maintenance for most chromosomes in the cell, which would explain the presence of high telomerase activity in all of the CML samples studied during BP [13]. This might also explain why some cells score positive for both telomere elongation mechanisms (Figure 3A): these are cells that are transitioning between both such that they have overlapping ALT and telomerase expression.

## Conclusion

In summary, this study is the first (to our knowledge) to demonstrate in CML during CP a high frequency of circular ECTR (specifically C-circles), one of the defining hallmarks of ALT pathway activation. The presence of ALT in CML during the CP strongly suggests that ALT might be required at the early stages of leukemogenesis, potentially by conferring a necessary proliferative advantage. The fact that later stage CML cells always display activated telomerase [24] suggests a model in which CML disease state progresses from ALT to telomerase in order to preserve telomere length and might reflect a transition between chronic and blast crisis phases. It will be important to determine how other cancers of given disease state or mutational status governs telomere length maintenance mechanisms. Clearly, these results will have a significant impact in the development of anti-telomerase based chemotherapies in cancer, since the presence of ALT will be an important determinant for drug resistance ultimately hindering patient treatment outcome.

## Methods

### Cell samples

Primary leukemia cells from blood or bone marrow of 24 CML patients in chronic phase (CP) were obtained from the Quebec Leukemia Cell Bank (Research Centre, Hôpital Maisonneuve-Rosemont, Montreal, Quebec, Canada). The diagnoses for all patients were confirmed by the presence of the Philadelphia (Ph) chromosome as the sole abnormality using conventional cytogenetics and fluorescence in situ hybridization (FISH) techniques before any specific treatment in the Quebec Leukemia Cell Bank.

### Telomerase activity

Telomerase activity was measured for each sample using the Quantitative Telomerase Detection Kit (Allied Biotech Inc., Germantown, MD, USA) according to the manufacturer's protocol, which is based on a PCR-based telomeric repeat amplification protocol. Briefly, 1×10^6^ cells were washed once in 1x PBS and then centrifuged at 12,000 g for 2 min at 4°C. The cell pellet was resuspended in 200 μL of lysis buffer (provided by the kit). Following incubation on ice for 30 minutes, the cell pellet was re-centrifuged at 12,000 g for 30 min at 4°C. Supernatant containing 100 ng of total protein from each sample extract was used to detect for telomerase activity. As a negative control, an equal amount of sample (100 ng) was heat inactivated at 80°C for 15 min. The positive control (provided by the kit) was used to generate a standard curve using six 5-fold serial dilutions which ranged in concentration from 0.5 pM to 0.00016 pM. Each serial dilution was amplified in a 25 μL PCR reaction containing 12.5 μL 2x QTD Premix (provided by the kit), 1 μL of the diluted control template and 11.5 μL H_2_O. For each sample (including both positive and negative control) a similar 25 μL reaction mix was prepared, using 12.5 μl 2x QTD Premix and 100 ng of total protein from each sample adjusted to a total volume of 25 μL with H_2_O. The PCR amplification was done on a Rotor-Gene 3000 qPCR machine (Corbett Life Science) using a 2-step reaction with the following parameters: an initial telomerase reaction at 25°C for 20 min followed by a PCR reaction cycle consisting of an activation step at 95°C for 10 min and 35 cycles of 1) a denaturation step at 95°C for 30 s, 2) an annealing step at 60°C step for 30 s, and 3) an extension step 72°C for 30 s. We plotted a standard curve using the C_t_ values and the concentration of the serial dilutions of the control template, from which we interpolated the activity of telomerase activity for each CML relative to the normal controls. All samples and controls were tested in triplicate. Telomerase activity of each CML sample was expressed as fold-increase to the average telomerase activity of three normal samples. An arbitrary cut off to differentiate low telomerase activity from high telomerase activity was defined as the lower bound of the 95% confidence interval of the average fold-increase calculated from all CML samples.

### ECTR detection

In order to assess the involvement of ALT in these CML samples we scored for the presence of circular ECTR by resolving telomeric-C and/or G-strands using 2D gel electrophoresis following a protocol previously reported by others [22] with minor modifications. Briefly, genomic DNA was extracted from cryopreserved blood or bone marrow of all CML samples and digested with HinfI and RsaI (New England Biolabs, Ont, Canada). The digested DNA was electrophoresed in the first dimension on a 0.4% agarose gel at 1 V/cm and at 4°C for a period of about 20 hours. Then, gel slabs from appropriate lanes were excised and run in a 1% agarose gel in the second dimension at 5 V/cm and at 4°C for a period of about 10 hours, which depended upon the gel apparatus used and the size of the specific telomere under study. The DNA was subsequently blotted onto nylon membrane in 20X SSC. The hybridization and detection of the telomeric DNA were accomplished by following our previous terminal restriction fragment (TRF) protocol [14] using digoxigenine labeled (CCCTAA)_3_ and (TTAGGG)_3_ probes (IDT, San Jose, CA) to detect the telomeric G- and C-strands, respectively. The local research ethic committees of all involved institutions approved the protocols used in this study and informed consent was obtained from each donor.

## Findings

In chronic myeloid leukemic at the chronic phase, an important proportion of cells presented extra chromosomal telomeric circles (specifically C-circles), hallmark of alternative lengthening of telomere, when telomerase is either present or not.

## Competing interest

JY is the principal investigator and takes primary responsibility for the paper. The authors reported no potential conflicts of interest.

## Authors’ contribution

OS performed the laboratory work for this study. AM participated in the data analysis and editing of the manuscript. JH recruited the patients. OS and JY wrote the paper. All authors read and approved the final manuscript.
